# Visible-Light Camera Sensor-Based Presentation Attack Detection for Face Recognition by Combining Spatial and Temporal Information

**DOI:** 10.3390/s19020410

**Published:** 2019-01-20

**Authors:** Dat Tien Nguyen, Tuyen Danh Pham, Min Beom Lee, Kang Ryoung Park

**Affiliations:** Division of Electronics and Electrical Engineering, Dongguk University, 30 Pildong-ro 1-gil, Jung-gu, Seoul 100-715, Korea; nguyentiendat@dongguk.edu (D.T.N.); phamdanhtuyen@dongguk.edu (T.D.P.); mblee@dongguk.edu (M.B.L.)

**Keywords:** visible-light camera sensor-based presentation attack detection, face recognition, spatial and temporal information, stacked convolutional neural network (CNN)-recurrent neural network (RNN), handcrafted features

## Abstract

Face-based biometric recognition systems that can recognize human faces are widely employed in places such as airports, immigration offices, and companies, and applications such as mobile phones. However, the security of this recognition method can be compromised by attackers (unauthorized persons), who might bypass the recognition system using artificial facial images. In addition, most previous studies on face presentation attack detection have only utilized spatial information. To address this problem, we propose a visible-light camera sensor-based presentation attack detection that is based on both spatial and temporal information, using the deep features extracted by a stacked convolutional neural network (CNN)-recurrent neural network (RNN) along with handcrafted features. Through experiments using two public datasets, we demonstrate that the temporal information is sufficient for detecting attacks using face images. In addition, it is established that the handcrafted image features efficiently enhance the detection performance of deep features, and the proposed method outperforms previous methods.

## 1. Introduction

To recognize a person, two major conventional recognition methods have been used in applications: token-based (keys, cards) and knowledge-based method (passwords) [1,2]. However, these techniques are inconvenient for users, because they must carry a key (or card), or remember a long and complex password identification. In addition, the key or password might be easily stolen by attackers. To overcome this problem, the biometric recognition technique can be used as an alternative. By definition, biometrics recognition is a recognition technique that uses one or more physical or behavioral characteristics of the human body to recognize/identify a person [1]. For this purpose, the biometric recognition technique only utilizes the unique body features for each individual for recognition. Several biometric recognition systems based on face, fingerprint, finger-vein, and iris data have been well studied and have various applications in daily life including airports, immigration offices, and companies, and smart phones [1,3,4,5,6,7,8,9]. Because physical/behavioral characteristics of a human are inherent features, this kind of recognition technique offers convenience to users. In addition, as shown in many previous studies, this recognition technique offers a very high recognition performance and its data are difficult to be stolen, compared with the conventional recognition methods [1,2].

Face recognition is one of the most popular biometric recognition methods used in various applications [8,9,10,11,12]. As inferred from the name, this method uses the human face data to distinguish a person from others. As shown in many previous studies, the face recognition system has been deeply studied and its recognition performance is now comparable or superior to human recognition, and is robust to changing capturing conditions [8,9,10,11]. However, this recognition method has recently been confronted with attacks in which a perpetrator can use a fake sample to successfully circumvent the face recognition system, instead of using the real face data. Therefore, the security of the recognition system is compromised, emphasizing the need to detect these kinds of fake samples before they can be used for recognition.

In previous studies, researchers demonstrated that the spatial information (information extracted using texture information from a still image) and temporal information (information extracted using the temporal dependency of images in an image sequence) are sufficient for a presentation attack detection (PAD) for a face recognition system (face-PAD) problem. However, they only used either spatial information or temporal information for face-PAD [13,14,15,16,17,18,19,20,21,22,23,24,25,26,27,28]. To overcome this problem and enhance the detection accuracy of face-PAD, our study proposes a new method for face-PAD that is based on a combination of both spatial and temporal information.

The remainder of this paper is organized as follows: Related studies on face-presentation attack detection (face-PAD) are provided in Section 2, and the contributions of our research are explained in Section 3. In Section 4, a detailed description of our proposed face-PAD method is provided, i.e., the preprocessing steps, the deep and handcrafted image feature extraction, and the classification using the support vector machine (SVM) method. In Section 5, we illustrate the results of intensive experiments using two public datasets, namely, the Institute of Automation, Chinese Academy of Sciences (CASIA) [13] and Replay-mobile [14], to validate the performance as well as demonstrate the efficiency of our proposed method over previous methods through a comparison of detection performance. Finally, we conclude our work in Section 6.

## 2. Related Works

To prevent attackers from accessing a face recognition system, several methods have been proposed to detect fake samples before they can be used as input to the system. Research on this purpose, namely face-PAD, normally extracts the discriminative features from input face images to distinguish a real image from a presentation attack (PA) image [15,16,17,18,19,20,21,22,23,24,25,26,27,28]. Then, a classification method such as a discriminative model or SVM is employed to classify the input images into real and presentation attack classes. These studies can be grouped into two: a study group that uses a still (single) image, and a study group that uses a sequence of images for detection. 

Most previous studies belong to the first group that uses a still image for face-PAD. One of the earliest studies in this group is that conducted by Tan et al. [16]. In their study, they classified real and presentation attack face images using a sparse low-rank bilinear discriminative model based on image features extracted by logarithmic total variation or a difference of Gaussian (DoG) methods. Through experimental results with an open dataset, namely, Nanjing University of Aeronautics and Astronautics (NUAA), they illustrated that it is possible to discriminate real and presentation attacks using face images. However, the detection performance of this study was not high because of weak image feature extraction. To extract adequate image features for a face-PAD system, Maatta et al. [17] employed three handcrafted image feature extraction methods, i.e., the Gabor filter, local phase quantization, and local binary pattern (LBP). With the extracted image features, face-PAD was performed using the SVM method. Due to a more powerful feature extraction method, the study by Maatta et al. [17] demonstrated a superior detection performance to the study by Tan et al. [16] using the NUAA dataset. Similar to the study by Maatta et al. [17], Benlamoudi et al. [22] applied the LBP method on local image regions (image patches) to extract image features. By dividing the entire face image into several image patches for extracting image features, the work by Benlamoudi et al. [22] extracted image features locally, which is sufficient to address the PAD problem because the fake features appear non-uniformly in a face image. Based on this approach, they showed a very high detection performance using the NUAA dataset. In a recent method proposed by Parveen et al. [23], a new feature extraction method, namely, dynamic local ternary pattern (DLTP), was applied for feature extraction. Using the SVM method for classification, they proved that DLTP works better than several feature extraction methods such as LBP or DoG.

Besides texture features, several other studies use the appearance features for face-PAD. In a study conducted by Boulkenafet et al. [19], the texture and color information were used for detecting presentation attack face images. This study is based on the fact that the color information can be changed owing to the effect of recapturing procedure. Therefore, color information can be used to discriminate the real and presentation attack face images. For this purpose, they first transformed the RGB image into the YCbCr color space to extract the color information of input image; then, they applied the LBP method to each individual channel of the color image in YCbCr space to extract the image features. The classification with the SVM method using the extracted color-LBP features with two open datasets, namely, CASIA and Replay-attack, confirmed the efficiency of this approach. In another study, Kim et al. [15] used the effects of defocus of face to discriminate real and presentation attack images. This study is based on the fact that the real face images are captured directly on a 3-dimensional face, whereas the presentation attack images are captured on a flat 2-dimensional face. Consequently, some parts of a real face such as nose and ears are relatively far from each other. Therefore, a case exists where, if the nose is in focus, the ear might be blurred, and vice versa, because of the narrow depth of field of the capturing device. Through experiments, the authors proved that defocus can be used for face-PAD. However, because this study using the defocus effect for face-PAD is based on the assumption that the presentation attack images are captured from a flat 2-dimensional face (print, video display), this method might to be difficult to be applied in the cases where 3-dimensional masks are used as presentation attack samples. Galbally et al. [21] proposed the use of image quality measurements for the face-PAD problem. In their study, they used 14 image quality measurements such as peak signal to noise ratio, image correlation, and total edge differences for feature extraction, and demonstrated a detection performance comparable to state-of-the-art methods using CASIA and the Replay-attack dataset.

Recently, with the development of deep learning-based methods such as the convolutional neural network (CNN), several studies have used this technique to address the face-PAD problem. Menotti et al. [26] used the CNN method for PAD for iris, face, and fingerprint recognition systems. To efficiently detect the presentation attack samples, they applied two optimization procedures, i.e., the architecture optimization and filter optimization, to optimize both the network architecture and its parameters. The results of this study demonstrated that the deep learning framework is sufficient for PAD, because it offers a better detection performance than the conventional methods based on handcrafted features. In a recent study by De-Souza et al. [20], they extracted deep texture features from face images by integrating the LBP descriptor to a modified CNN network for face-PAD. By integrating the LBP descriptor to a CNN network, this study acquired the advantages of both LBP and CNN to enhance the detection performance of the face-PAD system. However, the use of the deep learning-based method generally presents the problem of over-fitting or under-fitting due to the huge number of network parameters that are required to be learnt, from limited training data. To address this problem, a study by Nguyen et al. [27] combined the deep features extracted using the CNN method with handcrafted image features extracted using multi-level local binary pattern (MLBP) with SVM for the face-PAD problem. As a result, they demonstrated a higher detection performance than conventional systems that only use deep or handcrafted image features.

Although the above-mentioned methods have demonstrated a good detection performance for the face-PAD problem, they utilized texture information extracted from a single image. From observation, fake features can occur non-uniformly in a sequence of PA images captured in a certain period of time compared with a still image. This phenomenon can be caused by the change of illumination or pose of presentation attack instrument (PAI) during the image acquisition procedure that results in a difference between real and PA image. As a result, some still images could exist in a sequence that contains more discriminative information than the others. In simple words, we can extract more discriminative information for face-PAD using a sequence of images than from a still image. In a study by Xu et al. [28], they used a sequence of face images for face-PAD based on deep learning architecture by combining CNN and long-short term memory (LSTM), a special kind of recurrent neural network (RNN). They confirmed that the performance of the sequence-based method is higher than that of the still image-based method. However, this study used a very shallow CNN network with two convolutional layers and one fully connected layer for extracting image features. In addition, they used dense-connection as a classifier for face-PAD. This approach usually has a problem of over-fitting caused by a huge number of system parameters. 

Recently, thermal image-based and depth-image-based face recognition systems have been proposed in addition to the visible-light face recognition systems [29,30]. The use of these special imaging devices can help reduce the negative effects of illumination on face recognition systems. However, most face recognition systems in application including mobile devices use only the visible-light camera. In addition, the use of thermal or depth image cameras for face recognition systems has a limitation of the price of hardware of the systems. Higher cost of thermal or depth cameras than a conventional visible-light camera normally causes price increases for the recognition system, and consequently makes it hard to be applied in broad applications. Because of these reasons, our study only focuses on face-PAD for visible-light face recognition systems and we believe that our study provides a case for real applications at present. In Table 1, we summarize and compare previous studies on the face-PAD problem with the proposed method.

## 3. Contributions

Our research is novel in the following four ways compared with previous studies on the problem of PAD for face recognition.

- First, our study extracts the temporal information from successive input face images for detection, instead of using single images. Because a sequence of face images is used, not only is the extraction of richer information from inputs made possible, but the relation between each image in a given sequence is learnt to precisely detect the PA image.
- Second, inspired by the success of the CNN-based method for a computer vision system, we use a very deep CNN network to efficiently extract image features for each image in a given sequence images of faces. With the extracted image features, we use a special kind of neural network, named RNN, to learn the temporal information of the entire input sequence. This kind of network, named stacked CNN-RNN, allows our system to efficiently detect the PA sample using a sequence of faces.
- Third, we additionally extract handcrafted image features based on our proficient knowledge of the face-PAD problem using an extraction method named MLBP to enhance the performance of detection system. By combining the deep and handcrafted image features, we demonstrate that the performance of face-PAD system is significantly enhanced compared with the use of single deep or handcrafted image features.
- Finally, we make our proposed algorithm accessible [31] for reference and comparison for future studies on face-PAD problem.

## 4. Proposed Method

In this section, we explain the proposed method for face-PAD, including the architecture of stacked CNN-RNN network for deep image feature extraction, the handcrafted image feature extraction by MLBP method, and the classification method using SVM based on the extracted image features.

### 4.1. Overall Design of Proposed Method

**Figure 1:**
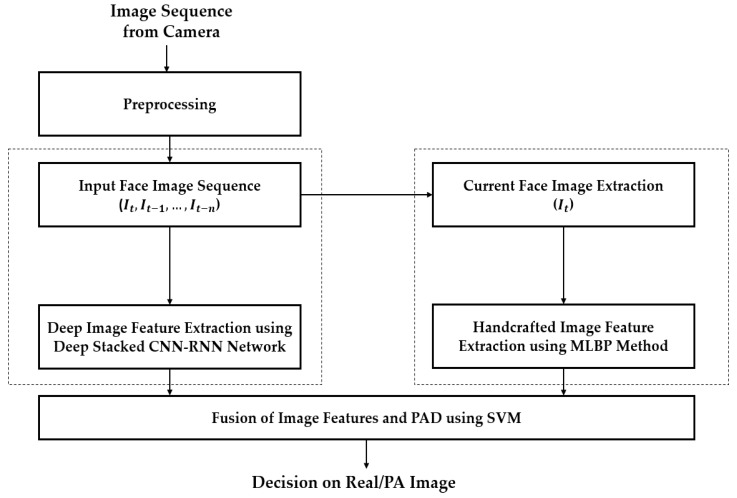
Working sequence of the proposed method for face-PAD.

In most previous studies, a still face image was used for face-PAD [16,17,18,19,20,21,22,23,24,25]. However, our observation illustrates that a difference between real and presentation attack images can not only appear in a single still image but also in a sequence of successive images. Fake features can occur in presentation attack images clearer in specific frames than in other frames, due to the effect of a change of illumination or the pose of a presentation attack instrument (PAI) during image acquisition. In addition, a conventional face recognition system can acquire and process a sequence of successive images instead of a single image to enhance the recognition performance. Therefore, the use of a single image could limit the performance of the face-PAD system. From these observations, we design a new detection method that is based on the image features extracted from both a current single image and a sequence of successive images, as shown in Figure 1.

As shown in Figure 1, the proposed method uses two kinds of image features for face-PAD, i.e., the deep features extracted by a stacked CNN-RNN network and handcrafted features extracted by the MLBP method. The proposed method acquires a sequence of images from capturing devices (camera). With a sequence of images, we first perform preprocessing to detect and align the faces. Details of this step are explained in Section 4.2. As a result of this step, we obtain a sequence of faces, including the current face and several faces from previous frames, as shown in Figure 1. Using this sequence of faces, we use a stacked CNN-RNN network to extract the associated temporal information. In addition to deep features, we also extract the texture features of the current frame using the MLBP method, which is further discussed in Section 4.3 and Section 4.4. As the final step of our proposed method, the extracted features (deep and handcrafted features) are used for classifying the input image sequence into either real or presentation attack classes using the SVM method.

### 4.2. Face Detection, Alignment, and Face Region Image Extraction

**Figure 2:**
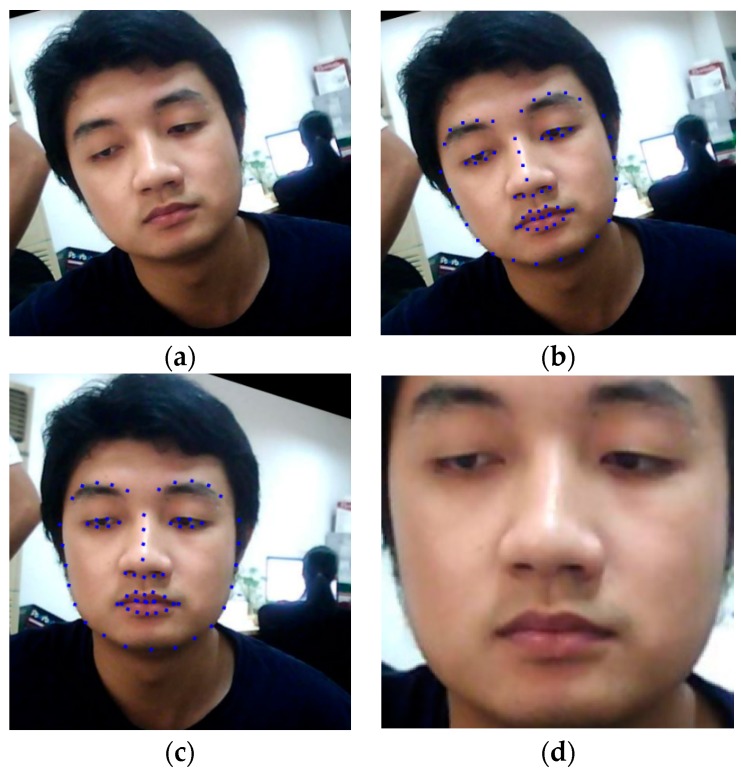
Demonstration of our preprocessing step: (**a**) input face image from NUAA dataset [16]; (**b**) detected face region on input face image using ERT method; (**c**) face region is aligned using center points of face, left and right eyes; (**d**) final extracted face region.

Similar to conventional face recognition systems, the initial step of the proposed method is the extraction of faces from an input image sequence. This step is essential for any face-based biometrics system that has the purpose of removing the background region that does not contain sufficient information [27]. Because this is a preprocessing step and not our main contribution, we use an efficient existing method proposed by Kazemi et al. [32] named ensemble of regression tree (ERT) to efficiently localize the face and its landmark points. Although other face detection methods exist, such as the adaptive boosting (Adaboost) that uses SVM on Haar-like or the LBP features [33] for face detection, the ERT method offers an important advantage: this method provides not only the face location, but also the landmark points. These landmark points are sufficient for indicating the face orientation and are useful for aligning faces. As indicated by a previous study by Benlamoudi et al. [22], face alignment plays an important role in the performance of the face-PAD system. In detail, we detect a total of 68 landmark points for a face as shown in Figure 2b,c. Using the detected landmark points, our study finds three special points, i.e., two center points of the left and right eyes, and one center point of the entire face region. These points can be used as rough indicators of face orientation. Based on these special points, we align a face by rotating the entire face region around the center point of the face, as shown in Figure 2b,c. We rotate the original face region, as shown in Figure 2b, by a rotation angle that is calculated using Equation (1) to obtain an aligned face region, as shown in Figure 2c. In this equation, (R_x_,R_y_) and (L_x_,L_y_) indicate the center points of the right and left eyes, respectively.

(1) $$\theta =tan^{-1}\left(\frac{R_{y}-L_{y}}{R_{x}-L_{x}}\right)$$

As a result, all the faces are aligned at the same center position and frontal view, instead of the natural orientation. As the final step of our preprocessing method, we crop the face using the largest bounding box of the face on the rotated face image and obtain the final extracted face region for further processing steps, as shown in Figure 2d.

### 4.3. Stacked CNN-RNN Architecture for Learning Temporal Information from Successive Images

The deep learning framework has been successfully employed in many computer vision tasks such as object detection [34,35], image classification [36,37,38], and image feature extraction [6,39]. Inspired by the success of this technique, our study uses a special deep learning network, named the stacked CNN-RNN, to learn and extract the temporal information from a sequence of face images.

**Figure 3:**
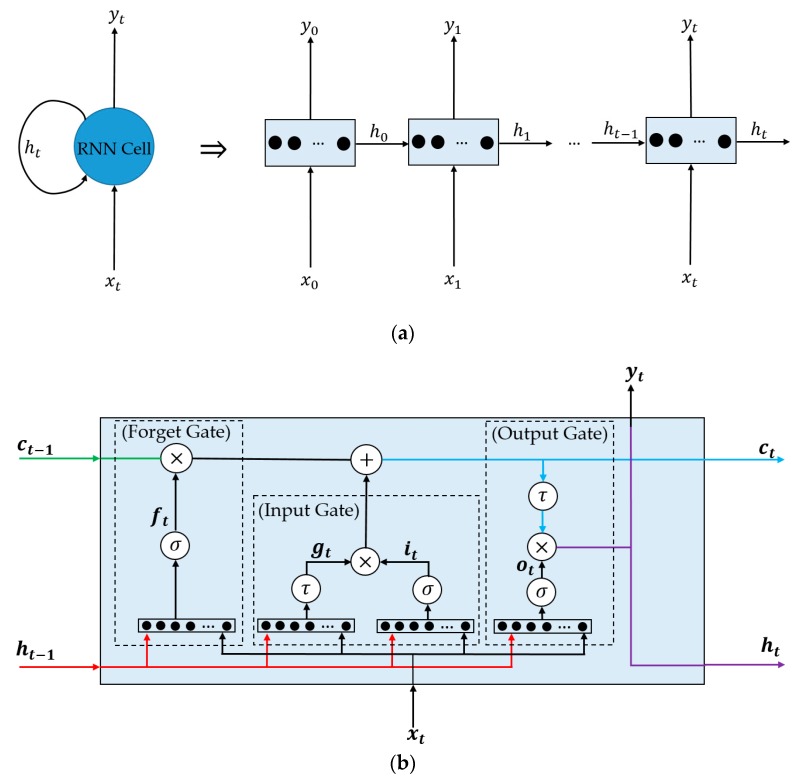
Demonstration of an RNN network: (**a**) a simple RNN cell; (**b**) structure of a standard LSTM cell.

To extract the deep image features for computer vision systems, several previous studies [6,39] have used the CNN networks. The use of a CNN network offers an important advantage over conventional handcrafted image feature extraction methods that the CNN network can learn to extract sufficient image features using a large amount of training data. Consequently, the performance of computer vision systems that use CNN is usually better than that based on conventional handcrafted image feature extraction methods. However, the CNN network also has its own limitations. Beside the limitations on the internal structure of its architecture, such as the over-fitting problem caused by the lack of training data and the huge volume of the network’s parameters, the CNN networks normally work with a single image to extract its texture features. As a result, it is prevented from learning temporal information from a sequence of images. To overcome this limitation, the RNN architecture is considered [40]. In Figure 3, the basic architecture of the RNN network is shown, to demonstrate its ability of learning the temporal information. Figure 3a shows the basic RNN memory cell (on the left) and its unrolled version (on the right). As shown in this figure, the RNN cell is a neural network that comprises several states. At the initial state (t = 0), the input to the RNN cell includes only input feature vector $x_{0}$, and the network will learn the output $y_{0}$ and a state vector $h_{0}$, as denoted in Equations (2) and (3). The state vector serves as a memory that stores some information of this state, and forwards the information to the next state of RNN cell. In these equations, f_01_ and f_02_ denote the functions learnt by the RNN network at the initial state using neural network. In a simple case of the RNN network, y_0_ and h_0_ are set to be equal.

(2) $$y_{0}=f_{01}\left(x_{0}\right)$$

(3) $$h_{0}=f_{02}\left(x_{0}\right)$$

As a result, at a certain state (t≠0), the input of an RNN cell includes the input feature vector $x_{t}$ and additional information $h_{t-1}$ that was produced by the RNN cell at stage (t − 1), as shown in Equations (4) and (5). As shown in Figure 3a and Equations (2)–(5), it can be seen that the output of a certain state (y_t_) is a function of not only the current input (x_t_), but also the memory obtained from previous state (h_t−1_). As shown in Equations (3) and (5), the state vector h_t−1_ is also a function of the previous input feature vector x_t−1_ and previous state h_t−2_, and so on. Consequently, the output y_t_ of RNN cell at state t contains information of not only the current input feature vector, but also the information of all previous states. Because of this reason, the RNN cell is called a memory cell.

(4) $$y_{t}=f_{t1}\left(h_{t-1},x_{t}\right)$$

(5) $$h_{t}=f_{t2}\left(h_{t-1},x_{t}\right)$$

Figure 3a and Equations (2)–(5) demonstrate the simple case of the RNN network. To utilize the RNN architecture more efficiently, our study employs a special variant of the RNN network, named long-short term memory (LSTM) [41]. This architecture is demonstrated in Figure 3b and is a sufficient design for the RNN to learn information for both long- and short-term inputs. For this purpose, the state vector of LSTM is divided into two parts: short-term memory ($h_{t}$) and long-term memory ($c_{t}$). For implementation, the LSTM architecture includes three gates, namely, the input, forget, and output gates, as shown in Figure 3b and Equations (6)–(12). In these figures and equations, σ indicates the standard sigmoid function that nonlinearly scales the input to the output in the range of 0~1; τ indicates the standard tanh function that scales the input nonlinearly to the output in the range of −1~1. Using the forget gate, the network will learn how much of long-term information is to be erased/retained for using in the current state. The input gate decides which information of this state is to be added to the long-term state. By combining the outputs of forget and input gates, the LSTM cell updates the long-term memory state, which verifies if the amount of information from previous states is sufficiently used for the current state. Finally, the output gate combines current input with the state vector to produce the output of the network. This kind of RNN network has been widely utilized in applications for gait recognition [42] and action recognition [43].

(6) $$i_{t}=\sigma \left(f_{it}\left(x_{t},h_{t-1}\right)\right)$$

(7) $$g_{t}=\tau \left(f_{gt}\left(x_{t},h_{t-1}\right)\right)$$

(8) $$I_{t}=\;g_{t}\times i_{t}$$

(9) $$f_{t}=\sigma \left(f_{ft}\left(x_{t},h_{t-1}\right)\right)$$

(10) $$o_{t}=\;\sigma \left(f_{ot}\left(x_{t},h_{t-1}\right)\right)$$

(11) $$c_{t}=f_{t}\times c_{t-1}+I_{t}$$

(12) $$y_{t}=h_{t}=\;o_{t}\times \tau \left(c_{t}\right)$$

**Figure 4:**
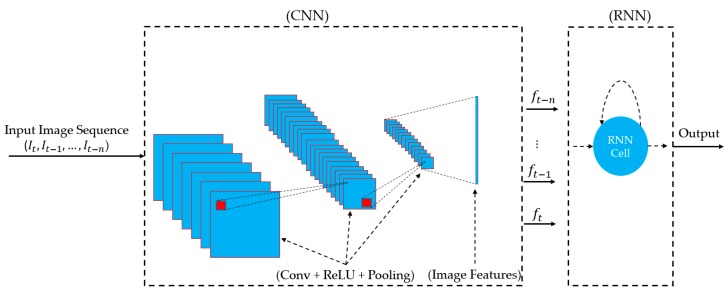
General architecture of a stacked CNN-RNN network for temporal image extraction.

As shown in Figure 3, the inputs of RNN cells are a sequence of image features that are extracted from a sequence of input images (in our study). In our study, we use the CNN network to extract sufficient image features for the RNN network. As a result, we constructed a stacked CNN-RNN network architecture, as shown in Figure 4. In Table 2, we describe in detail, the architecture of the stacked CNN-RNN network shown in Figure 4. As shown in Table 2, our stacked CNN-RNN network includes two parts of CNN and LSTM stacked together. In detail, the CNN part uses the convolutional layers from VGG-19-Net that is responsible for image feature extraction using the convolution operation [36]. Although it is possible to use other CNN network architectures, we use VGG-19-Net in our study for a certain choice because our study focuses on the temporal information extraction using stacked CNN-RNN architecture, not on the CNN. In original VGG-19-Net, the outputs of the convolutional part are 512 feature maps with the size of 7 × 7 pixels. Consequently, the dimension of the extracted image features is about 25,088 (512 × 7 × 7). This number is too large to be used as an input for the RNN network. Therefore, we perform an additional global average pooling operation on the outputs of convolution layers of VG-19-Net. As a result, we obtain 512 features maps of 1 × 1 pixels. This indicates that the extracted features for each input image are a 512-dimensional vector that is much smaller than the 25,088-dimensional vector.

Because the extracted features by convolutional layers of CNN network are the abstract texture features, we further perform an additional manipulation on these features using a fully-connected layer with 1024 neurons, to convert the extracted 512-dimensional texture features to abstract 1024-dimensional image features. This fully-connected layer serves two purposes: First, we further learn the extracted image texture features to convert them to abstract features that are little affected by the characteristics of detail of texture information in images. Second, as shown in Table 2, we utilize a batch normalization layer in addition, to normalize the extracted features to reduce the overfitting problem and the difference between the training and testing images. As a result, we extract a sequence of normalized 1024-dimensional feature vectors of an image sequence and use them as the input of LSTM cell. In the final step of our design for the stacked CNN-RNN network, the output of the LSTM cell is connected to the output of the network. Because the purpose of our study is presentation attack detection, the output of our network has two neurons which will decide if the input image sequence belongs to “real” or “presentation attack” class. As shown in Table 2, we also use dropout method to reduce the overfitting problem that usually occurs with deep networks [44].

### 4.4. Handcrafted Image Feature Extraction Based on the MLBP Method

The deep features extracted by stacked CNN-RNN network, as mentioned in Section 4.3, are obtained by a learning procedure using two main operations of convolution and fully-multiplication using a large amount of training samples. However, there are several difficulties for this kind of deep neural network in extracting the optimal image features. One main difficulty is the under-fitting or over-fitting problems that generally occur with deep neural networks due to several reasons, such as lack of training data, and a huge number of network parameters. Because of these difficulties, although the deep neural network has been proven to be better than the conventional method for computer vision systems, it cannot be considered that the image features extracted by this method are optimal. Therefore, as suggested by several previous studies, our study extracts the handcrafted image features using the MLBP method, beside the deep features extracted by stacked the CNN-RNN network to obtain adequate information from the input image.

The local binary pattern was initially used as an image texture descriptor for image classification [45,46] and human age estimation [47]. In our previous study, this method was successfully used for face-PAD problem [27]. By definition, the local binary pattern method can be considered as an encoding method that encodes a pixel in the image using its surrounding pixels, as shown in Equation (13). As shown in Equation (13), the LBP method encodes a pixel into a sequence of P (bits) using P surrounding pixels and an adaptive threshold function. This method offers an important characteristic to the encoded image that the encoded image is invariant to the change of illumination.

(13) $$LBP_{R,P}=\sum_{i=0}^{P-1}2^{i}s\left(g_{i}-g_{c}\right)where\;s\left(x\right)=\;\{\begin{array}{c} 1\;if\;x\geq 0\\ 0\;if\;x<0\; \end{array}$$

Another important characteristic of the LBP method is that an LBP code of each pixel in image is sufficient to represent various micro-texture features such as edge, corner, blob, and line-end in image. Based on these two characteristics, we accumulate the histogram of micro-texture features over a given image, and use this histogram as an image texture features for face-PAD. The use of these kinds of texture features offers two advantages for face-PAD. First, the extracted image features are invariant to the change of illumination. Second, the histogram features can reflect the distribution of micro-texture features on face image. For the face-PAD problem, whereas the real images contain normal texture features, the presentation attack images can contain additional abnormal ones such as dot noise, broken textures, or blurring caused by the imperfection of presentation attack process. As a result, the distribution of micro-texture features is odd compared with those of real images. 

**Figure 5:**
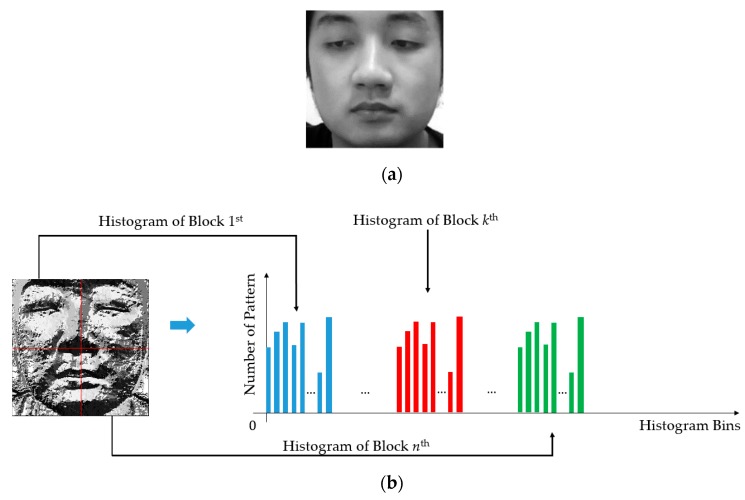
Handcrafted image feature extraction process using the MLBP method: (**a**) an input face image from NUAA dataset [16]; (**b**) formation of the MLBP features of (a) (left: encoded LBP image; right: LBP features).

To accumulate the histogram of micro-texture features, we first encode all the pixels in a given image using the LBP operator, as shown in Equation (13) to obtain an encoded image. Then, its pixels are classified into several categories according to specific kinds of micro-texture features, i.e., uniform and non-uniform patterns, with a definition that the uniform patterns contain at most two transitions from 0 to 1 (or 1 to 0), and the non-uniform pattern contains more than 2 transitions from 0 to 1 (or 1 to 0). Figure 5 shows the methodology of the LBP feature formation used in our study. In our experiment, we extracted the LBP features for different levels of the radius (R) and resolution (P) of the LBP operator to form a new feature, namely, multi-level LBP (MLBP), to capture rich texture information from each face image. In detail, we used three possible values of R (1, 2, and 3) and three values of P (8, 12, and 16) in our experiment. Consequently, we obtained a feature vector of 3732-component for each face image [27].

### 4.5. Presentation Attack Detection Using SVM

**Figure 6:**
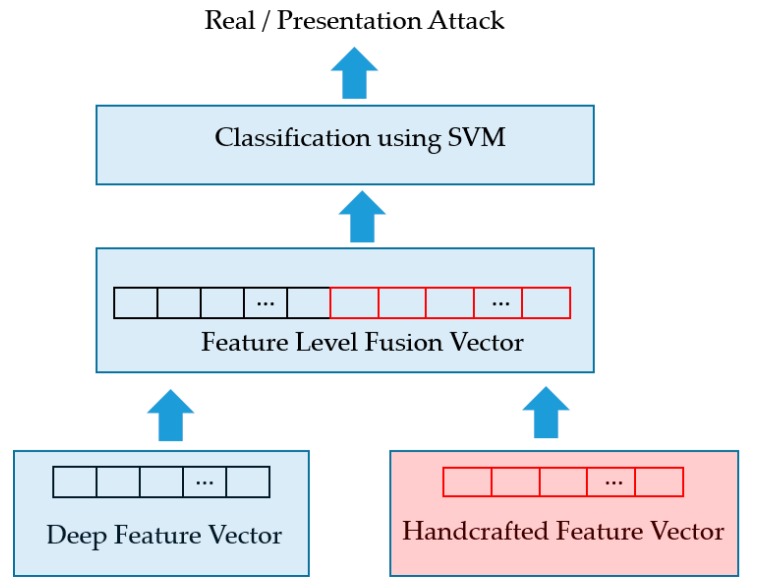
Feature level fusion approach.

Using the two feature extraction methods mentioned in Section 4.3 and Section 4.4, we extract two feature vectors for an input sequence of faces, i.e., a 1024-dimensional deep feature vector and 3732-dimensional handcrafted feature vector. As the final step of our proposed method, we employ the SVM method to classify the input sequence into a real or presentation attack class using these extracted feature vectors. For this purpose, our study utilizes two approaches for combining these two feature vectors, i.e., the feature level fusion (FLF) and score level fusion (SLF) [27,39]. As the first approach of feature level fusion, a concrete combined feature vector is formed by concatenating the two individual vectors. As a result, we obtain a new feature vector, named the feature level fusion vector, which is a 4756-dimensional vector. Because this new feature vector is a combination of the two extracted feature vectors, it contains information associated with each feature vector. This combined feature vector is used as the input to SVM for the purpose of classification. In Figure 6, we show the graphical demonstration of this combination approach.

**Figure 7:**
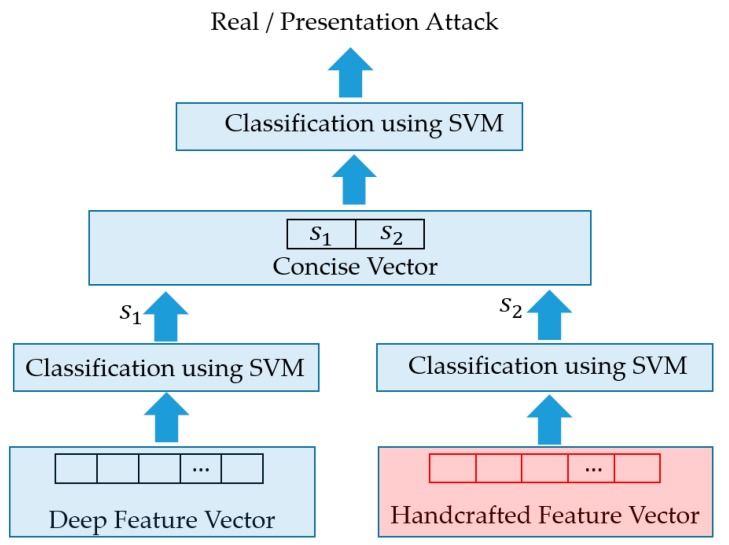
Score level fusion approach.

As the second combination approach, we first use the SVM method to classify the input sequence into a real or presentation attack class using the individual feature vector (deep or handcrafted vector). As the output of each classifier, we obtain an output value that represents the distance from the input vector to the classifier. In our study, this value is considered a new concise feature that represents the possibility of an input sequence belonging to real or presentation attack classes. As a result, we obtain two output values for two feature vectors. These output values are then concatenated to form a new 2-dimensional feature vector that is finally used as the input of additional SVM to classify the input sequence face as real or presentation attack classes. The graphical flow chart of this approach is depicted in Figure 7. 

As mentioned above, our study utilizes the SVM method for classification. This up-to-date classification method and has been widely used in many computer vision systems for classification or regression purposes [48]. This method uses a training dataset to construct the best suitable hyper-plane for separating two (or many) classes by maximizing the distance (called margin) from the selected classifier to several nearest data samples (called support vectors). For complex problems such as non-linear classification, the SVM method employs a special technique, called kernel function, to transform data from a low dimension space to a higher dimension space, on which the new data can be easily separated by a hyper-plane. By definition, the SVM constructs a hyper-plane by selecting several support vectors, as shown in Equation (14). In this equation, **x_i_** and **y_i_** are the selected support vectors and their corresponding labels (−1 or 1), a_i_ and b are the parameters of the SVM model, and **K()** is the kernel function, as mentioned above. In our experiments, we use three common kernel functions, i.e., the linear kernel, radial basis function (RBF) kernel, and polynomial kernel, as shown in Equations (15)–(17).

(14) $$f\left(x\right)=\overset{k}{\underset{i=1}{\sum }}a_{i}y_{i}K\left(x,x_{i}\right)+b$$

(15) $$\;\mathrm{Linear}\;\mathrm{kernel}:\;K\left(x_{i},x_{j}\right)=x_{i}{}^{T}x_{j}$$

(16) $$\mathrm{Radial}\;\mathrm{basis}\;\mathrm{function}\;\mathrm{kernel}:\;K\left(x_{i},x_{j}\right)=e^{-\gamma \|x_{i}-x_{j}\|{}^{2}}$$

(17) $$\mathrm{Polynomial}\;\mathrm{kernel}:\;K\left(x_{i},x_{j}\right)={\left(\gamma x_{i}{}^{T}x_{j}+coef\right)}^{degree}$$

To reduce the complexity of training the SVM model as well as reducing the effect of noise, our study applies the principal component analysis (PCA) technique to reduce the dimension of input features to the SVM [27,39]. In our experiment, the number of principal components is selected based on the variance of projected data on all possible axes; that is, we select the number of principal components such that the total variance of selected axes is greater than 99% of total variance of all possible axes. For implementation, we use several python packages, including keras for implementing the deep neural network [49], and sci-kit learn for implementing the PCA and SVM [50] techniques.

## 5. Experimental Results

### 5.1. Experiment Setups

Based on our design, the proposed method receives a sequence of face images to decide which class that input sequence belongs to. In our experiment, we set the size of sequence (number of images) to five images. In addition, we collect five images at the interval of 12 frames to form an image sequence. This setup is selected to enable image collection with a large time difference, so that the images in sequence exhibit a large difference, allowing our algorithm to work properly. As mentioned in Section 4, our study focuses on extracting spatial and temporal information from sequence of images for face-PAD. Therefore, the length of sequence plays an important role in the system performance. With the short sequence length, the temporal information is low, but the long sequence length increases the processing time and the effects of noise. We experimentally determined these optimal values (five images) by considering both the effect of noise and processing time of face-PAD system. 

As shown in Section 4, our proposed method combines the two kinds of image features for face-PAD, i.e., the deep features extracted by a deep stack CNN-RNN network, and the MLBP features. To train the stacked CNN-RNN network, we employed the stochastic gradient descent (SGD) optimizer method. In addition, we initialized the network parameters of the CNN using a pre-trained VGG-19-Net model, which was successfully trained on the ImageNet dataset [36,49]. This scheme has been used in previous studies to initialize the network parameters well, reduce the training time, and consequently make the network easier to train. In Table 3, the parameters of training procedure used in our experiments are specified. Our algorithms were implemented using Python language, and all the experiments including training and testing were performed in a desktop computer with Intel Core i7 CPU (Intel Corporation, Santa Clara, CA, USA), working at 3.4 GHz, 64 GB of RAM memory, and Titan X graphics processing unit (GPU) [51].

To measure the performance of a PAD system, we followed the ISO/IEC30107-3 standard [52]. In detail, we use two metrics, namely, attack presentation classification error (APCER) and bona-fide presentation classification error rate (BPCER), to measure the performance of our proposed method as well as compare them with those reported by previous studies. By definition, the APCER is an error that occurs when an attack presentation is falsely accepted as a bona fide (real) image; and BPCER is the error rate that occurs when a bona-fide image is falsely rejected as the attack presentation image. These two measurements have trade-off characteristics. Therefore, we use an average measurement of the two, namely, the average classification error rate (ACER), to measure the overall performance of PAD system, as shown in Equation (18).

(18) $$ACER=\frac{APCER+BPCER}{2}$$

The APCER and BPCER metrics are analogous to the two common error measurements of a recognition system, namely, the false acceptance rate (FAR) and false rejection rate (FRR). However, the APCER and BPCER are measured for each type of attack according to each presentation attack instrument (PAI). As indicated in Equation (18), the low value of ACER indicates a small detection error and consequently a high performance of a PAD system. In addition to the APCE, BPCER, and ACER metrics, we also measure the half-total-error rate (HTER) by averaging the detection error without considering the type of attack, for comparison with several previous studies.

### 5.2. Description of Datasets

To evaluate the performance of our proposed method as well as compare it with those produced by previous studies, we use two open datasets, namely, the CASIA dataset [13], and Replay-mobile dataset [14]. These datasets have been widely used for evaluating the performance of face-PAD systems in previous studies. The CASIA dataset contains real and presentation attack attempts of 50 people with a large variation of the quality of face regions (low, normal, and high quality) using three presentation attack instruments of wrap-photo, cut-photo, and video display. For each person, three real access video files were collected according to three different quality of faces, and at each level of the quality of face, three presentation attack video files were captured according to three PAIs (wrap-photo, cut-photo, and video). Consequently, the CASIA dataset contains a total of 600 video files (150 video files for real access, and 450 video files for presentation attack). Because this dataset is open to research on face-PAD problem, it was pre-divided into two sub-sets of training and testing datasets, from which the training dataset is used to learn the face-PAD classifier/detector, and the testing dataset is used to measure the performance of classifier obtained using the training dataset. In Table 4, we show the description of the CASIA dataset and its sub-datasets. Using the face detection method mentioned in Section 4.2, we detected the face images for CASIA dataset from each video file. In addition, we artificially applied the data augmentation method on training dataset to generalize the training data, as shown in Table 4. This is a common method that helps to reduce the effect of the overfitting problem caused by the lack of training data in deep learning networks.

The second dataset used in our study (Replay-mobile) was constructed for the purpose of detecting a presentation attack for a mobile-based face recognition system [14]. This dataset contains a total of 1190 video files of real and presentation attacks attempts of 40 people under different lighting conditions using mobile devices (phone or tablet). Two presentation attack instruments were used, i.e., print photo and video display. Among 1190 video files, 1030 video files are used for face-PAD, whereas the other 160 video files of real access are used for measuring the performance of face recognition system. For a fair comparison between different face-PAD methods, the Replay-mobile dataset was pre-divided into three different datasets, namely, the training, validation, and testing datasets, without overlap between the datasets. Among these sub-datasets, the training dataset is used for training detection model, whereas the validation dataset is used to optimally select system parameters that could not be obtained using the training data, and the testing dataset is used to measure the performance of the detection system in general. 

In Table 5, the description of the Replay-mobile dataset used in our study is shown. Similar to the CASIA dataset, we utilized the face detection and alignment method presented in Section 4.2 to detect faces from video files and form the image sequences for our detection system, as shown in Table 5. In addition, we performed data augmentation on the training and validation datasets to generalize them and to reduce the effect of overfitting problem on our stacked CNN-RNN model during training. The above-mentioned datasets are large and have been widely used for face-PAD in previous studies. Because of this reason, our study uses these datasets to evaluate the performance of our proposed method, and compares it with various reported performances produced by previous studies.

### 5.3. Experimental Results

In this section, we present the experimental results using our proposed method in Section 4 with two public datasets mentioned in Section 5.2 (the CASIA and Replay-mobile datasets) 

#### 5.3.1. Experiment Using the CASIA Dataset

**Figure 8:**
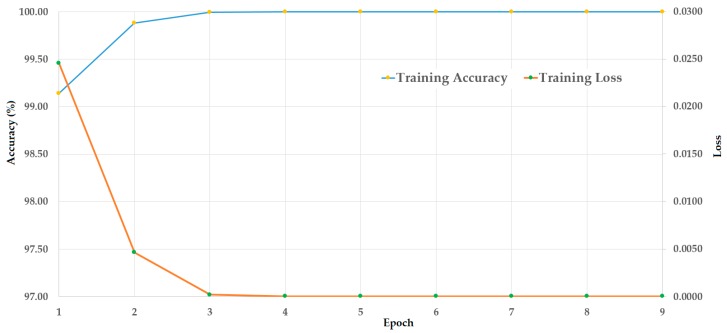
Convergence graphs (accuracy and loss) of the training procedure on the CASIA dataset.

In this experiment, we used the CASIA dataset to evaluate the detection performance of the proposed method mentioned in Section 4. We considered that the CASIA dataset was made of three different PAIs, i.e., wrap-photo, cut-photo, and video display, to simulate three possible attack methods based on wrap-photo, cut-photo, or video display. First, we trained the stacked CNN-RNN network mentioned in Section 4.3 using the training dataset. Because the CASIA dataset was pre-divided into two sub-datasets (training and testing), we only used the augmented training dataset presented in Table 4 for this experiment. The result of this experiment is shown in Figure 8. As seen from Figure 8, the training of the stacked CNN-RNN network was successfully performed by causing the loss to reduce to reach zero, and increasing the accuracy to reach 100%, with the increase of training epoch. 

With the pre-trained stacked CNN-RNN model, we continued performing experiments with our proposed method using the SVM on the extracted deep and handcrafted image features. Detailed experimental results are provided in Table 6 to be used for the testing dataset. In this table, we provided the experimental results for four system configurations, i.e., the face-PAD system only using deep features extracted using the stacked CNN-RNN network, the face-PAD system only using MLBP features, and our proposed approach using feature level fusion (FLF) and score level fusion (SLF). As explained in Section 4.3, most of previous studies used CNN for deep feature extraction. To demonstrate the influences of our architecture that uses RNN for image feature extraction instead of using only CNN architecture, we also provided the detection performance of face-PAD system that uses deep features by CNN in Table 6. For this purpose, we performed experiments using the method proposed by Nguyen et al. [27] in which the VGG-19 network architecture was invoked for deep feature extraction. However, as shown in Table 2, our approach uses a stacked CNN-RNN network to extract a 1024-dimiensional feature vector for each sequence of image while the work by Nguyen et al. [27] used the original VGG-19 network to extract a 4096-dimensional feature vector of each input image. The use of original network architectures as Nguyen et al. [27] in this experiment makes an unbalanced comparison because of the different size of extracted image feature vectors. Therefore, we reduced the number of neurons in the last two fully-connected layers of the VGG-19 network from 4096 to 1024 in our experiment. By using this set-up, we extract a same-size feature vector for an input of each network, and therefore, we can fairly compare the detection accuracy of the two network architectures. It can be inferred from Table 6 that the deep features outperform the MLBP features for the face-PAD problem. The face-PAD system based on deep CNN-RNN features produced errors (ACER) of 1.458%, 0.858%, and 1.108% for the use of wrap-photo, cut-photo, and video display, respectively. Because the wrap-photo produced the worst APCER value compared with cut-photo and video access, the final error of face-PAD system using deep CNN-RNN features is about 1.458%. As shown in the first row of Table 6, the face-PAD system based on deep features extracted only by CNN network [27] has an error of 3.373%. This error is much higher than the error produced by our face-PAD system based on deep CNN-RNN features. Through this experimental result, we observed the positive influence of RNN architecture over the CNN architecture for the face-PAD system.

**Figure 9:**
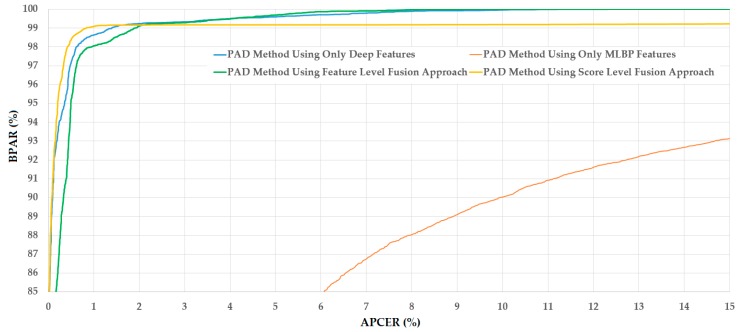
DET curves of the face-PAD systems using various feature combination approaches with a testing subset of the CASIA dataset.

Using only the MLBP features, we obtained the ACERs of 9.738%, 10.181%, and 9.884% for the use of wrap-photo, cut-photo, and video display, respectively. As a result, the overall error of the face-PAD system using only the MLBP features is about 10.181%. This error is much higher than that produced by the system that only uses deep CNN-RNN features (1.458% vs. 10.181%). As mentioned in Section 4.4, our study uses the MLBP method to extract spatial image features besides the deep features extracted by a stacked CNN-RNN network. By combining several LBP features at different scales and resolutions, we can extract more powerful image features than the conventional LBP method. Although the LBP method has been widely used for the face-PAD problem in previous research [17,19,22], it is still a handcrafted image feature extraction method. Therefore, it just captures limited aspects of the face-PAD problem. By definition, the LBP method is designed to capture texture (spatial) features on face regions by accumulating histograms of uniform and non-uniform micro-texture features such as edge, corner, blob, and flat regions. Because of this design, the LBP method can be affected by noise and/or background regions. As a result, the performance of LBP method is limited. As shown in a previous study conducted by Benlamoudi et al. [22], the LBP method produced a PAD error of about 13.1% using the CASIA dataset. In another study, Boulkenafet et al. [19] showed that the LBP features extracted from color face images work better than LBP features extracted from gray-scale face images. They showed a PAD error of about 6.2% with the CASIA dataset. These results are consistent with that (9.488%) in our experiments shown in Table 6, and they confirm that although the LBP features can be used for face-PAD, their performance is limited compared with the deep features. Although the performance of the face-PAD system using MLBP features is worse than that of the system using deep features, the use of both handcrafted and deep features of our study help enhance the detection performance of the face-PAD system. As shown in the lower part of Table 6, the score level fusion approach produced an overall error of about 1.286%, which is much smaller than 1.458% for the system using only deep features or 10.181% for the system using only handcrafted features. In addition, Table 6 shows the HTERs of 0.954%, 9.488%, and 0.910% for the face-PAD system that only uses deep features, only MLBP features, and score level fusion approach, respectively. This result again confirms that the combination of deep and handcrafted image features is sufficient to enhance the detection accuracy of the face-PAD system. This phenomenon is reasonable because deep and handcrafted feature extraction methods work on two different aspects (learning and non-learning). Therefore, they can complement each other and consequently enhance the performance of a face-PAD system. For demonstration, the detection error tradeoff (DETs) curves of the four system configurations used in this experiment are shown in Figure 9. In this figure, we drew the change of APCER according to the change of bona-fide presentation acceptance rate (BPAR). The BPAR is measured as (100 – BPCER) (%). As a result, the shape of DET curves are obtained as presented in Figure 9, and the curve at the higher position indicates better detection performance of the face-PAD system. As shown in this figure, the score level fusion approach outperforms the other configuration.

In some previous studies which used the CASIA dataset for performance evaluation, the detection performance was not only evaluated using the entire CASIA dataset, but also with several subsets of this dataset to validate the detection performance according to the quality of faces and type of attack samples [13,18,19,24,27]. Therefore, we performed similar experiments to evaluate the detection performance of our proposed method as well as compare the results with previous studies. For this purpose, we first divided the entire CASIA dataset into six subsets according to the quality of face regions and the type of attack method. As a result, we obtained six new datasets, i.e., “Low quality”, “Normal Quality”, “High Quality”, “Wrap-Photo”, “Cut-Photo”, and “Video Display”. Detailed descriptions of these datasets are provided in Table 7. For each sub-dataset, the training data and testing data are obtained by taking the corresponding data in the entire training dataset and testing dataset, respectively. Therefore, the training and testing dataset of each sub-dataset do not contain the overlapped data of the same people. Similar to the experiment with the entire CASIA dataset, we used the training data of each sub-dataset to train the classification model, and used the testing dataset to evaluate the detection performance. The detailed experimental results of this experiment are provided in Table 8. As shown in this table, we obtained the smallest detection errors (ACER) using our proposed method for all six sub-datasets. Among the six sub-datasets, we obtained the smallest detection errors of 1.417%, 0.004%, 1.085%, and 1.423% using the score level fusion approach for “Low Quality”, “Normal Quality”, “High Quality”, and “Video Display” datasets, respectively. For the “Wrap-Photo” and “Cut-Photo” datasets, we obtained the smallest errors of 1.886% and 0.425%, respectively, using the feature level fusion approach. However, as shown in Table 8, the difference between the feature level fusion and score level fusion for these two datasets is very small (0.119% for “Wrap-Photo” dataset and 0.003% for “Cut-Photo” dataset). From this result, it can be concluded that the proposed method with the score level fusion approach performs well with the CASIA dataset, and outperforms all previous studies using the same dataset.

As the final experiment in this section, we performed a comparison between the detection performances of our proposed method and those produced by previous studies. As shown in Table 9, the baseline method produced presented a detection error of about 17.000% [13]. This error decreased to 13.1%, 6.2%, 5.4% and 5.07% in later research [18,19,22,23]. In a recent study conducted by Nguyen et al. [27], they presented an error of about 1.696%. Compared with all of these detection accuracies, the proposed approach produced the lowest detection error. Based on this result, we conclude that our proposed method is sufficient for PAD for the face recognition system, and it is the state-of-the-art result obtained using the CASIA dataset.

#### 5.3.2. Experiment Using the Replay-Mobile Dataset

As shown in our experiments in Section 5.3.1, our proposed method demonstrated a better detection accuracy than other previous studies using the CASIA dataset. In our next experiment, we use an additional public dataset, namely, Replay-mobile, to evaluate the detection performance of our proposed method. The use of this additional dataset helps in the evaluation of the performance of our proposed method under various working environments of the face recognition system. This is a significantly large dataset that was collected for the purpose of detecting presentation attack face images for mobile devices. In our experiments, we grouped the presentation attack images into two different PAIs, i.e., matte-screen (photos and videos of people are displayed on a Philips 227ELH monitor, Philips, Amsterdam, Netherlands), and print (hard-copies of high-resolution digital photographs of people are printed on A4 matte paper). Different from the CASIA dataset, the Replay-mobile dataset was pre-divided into three subsets of training, validation, and testing. In this experiment, we used the training dataset to train the stacked CNN-RNN model for deep feature extraction, as well as the SVM model for final classification. The threshold for classification of an input face sequence into real or presentation attack classes is optimally selected using the validation dataset such that the real and presentation attack data are best separated. Finally, the performance of the detection system with actual data is evaluated using the testing dataset.

**Figure 10:**
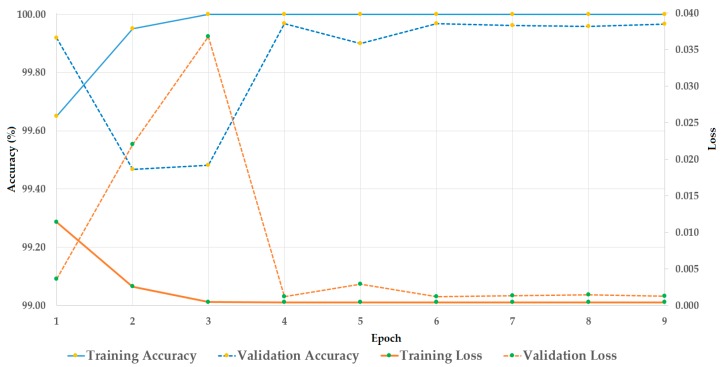
Convergence graphs (accuracy and loss) of the training procedure on the Replay-mobile dataset.

Similar to the experiments with the CASIA dataset, we first performed a training procedure to train the stacked CNN-RNN network for the deep feature extraction model. Figure 10 shows the result of this experiment. Because the Replay-mobile dataset also provides a validation set for validation purposes, we also measured the accuracy and loss of this dataset; these results are shown in Figure 10. The training procedure was successfully done using the training dataset by producing a classification accuracy of 100%, and causing the loss value to reduce to 0. Using the validation dataset, a similar result was obtained with a slightly lower performance than the case of using the training dataset.

In Table 10, the detection performance of five face-PAD system configurations using the Replay-mobile dataset is provided. In this table, the optimal threshold for real and presentation attack classifications is selected at the equal error rate (EER) point of the validation set. As shown in this table, the face-PAD system that only uses deep features extracted by our stacked CNN-RNN network produced an EER of 0.002%, and the face-PAD system that only uses the deep features extracted by CNN network [27] produced an error (EER) of 0.067% for the validation dataset. By applying the classification model to the testing dataset, we obtained the final detection errors (ACER) of 0.015% and 0.0045% using the deep features extracted by our stacked CNN-RNN and CNN networks, respectively. From these results, we can see that the RNN architecture is more efficient than the CNN architecture in extracting distinguish information from input face images. Using only the MLBP features, we obtained an EER of 4.659% for the validation dataset and a final ACER of 5.379% for the testing dataset. Similar to the experiment with the CASIA dataset, the detection performance using handcrafted features is worse than that produced using the deep features. However, the detection error was reduced to 0% for both validation and testing datasets using the feature level fusion approach. Using the score level fusion approach, the detection performance was maintained the same as that produced by the face-PAD system that only uses the deep features. However, as shown in Table 10, the error is very small and was caused by a single incorrect image sequence from the total of 32169 real sequences. From these results, we conclude that our proposed method performs well with the Replay-mobile dataset. The important reason that our proposed method works better with the Replay-mobile dataset than the CASIA dataset is that the CASIA dataset contains larger variation of a presentation attack scenario than the Replay-mobile dataset. As mentioned in Section 5.2, the CASIA dataset contains presentation attack images with three different quality of face images (low, normal, and high qualities), and three attack materials (wrap-photo, cut-photo, and video display), whereas the Replay-mobile dataset only contains the video display and print photo. Therefore, it is harder to detect presentation attack images when using the CASIA dataset than the Replay-mobile dataset. Because the detection error produced by this experiment was almost 0.000%, we do not show the DET curve for these experiments.

To demonstrate the efficiency of our proposed method, we compared our detection result with that of the baseline method. In the study conducted by Costa-Pazo et al. [14] (the author of Replay-mobile dataset), they presented an HTER of about 7.8% and ACER of about 13.64% using the image quality measurement (IQM) method, and an HTER of about 9.13% and ACER of about 9.53% using the Gabor-jets feature extraction method. It can be clearly seen that the detection errors of our method (0% using feature level fusion and 0.0015% using score level fusion approach) are much smaller than the errors produced by the baseline method reported by Costa-Pazo el al. [14]. This comparison demonstrates that our proposed method is sufficient for face-PAD and outperforms the previous studies using the same working dataset.

Because the deep learning-based method normally needs to use a huge amount of data to successfully train a network, it takes long time for the training procedure. The training time is mainly dependent on two factors, i.e., the network architecture (the amount of trainable parameters and the depth/wide of network) and the amount of training data. Using our proposed method and the CASIA dataset that contains 133936 image sequences for training (with data augmentation), it takes about 5 h per epoch. With the Replay-mobile dataset that contains 219011 training image sequences (with data augmentation), it takes about 7 h per epoch for training our network. As shown in Table 3, we trained the detection model using 9 epochs. Consequently, it takes about 45 h and 63 h for the CASIA and Replay-mobile datasets, respectively.

#### 5.3.3. Cross-Dataset Detection

In this experiment, we performed cross-dataset testing to evaluate the effect of difference in the image capturing conditions and setup. For this purpose, we performed experiments for two scenarios. In the first scenario, we trained the detection model using the CASIA dataset and validated the detection performance using the Replay-mobile dataset. In the second scenario, we exchanged the rule of the two datasets in the first experiment, i.e., we trained the detection model using the Replay-mobile dataset, and validated the detection performance using the CASIA dataset. 

As a result, we obtained the experimental results as shown in Table 11 and Table 12, for the first and second scenarios, respectively. For the first scenario, we obtained an HTER of 12.459% and ACER of 13.509% using the feature level fusion approach. Using the score level fusion approach, the errors increased to an HTER of 20.632% and ACER of 23.589%. For the second scenario, the errors were higher with an HTER of 42.785% and ACER of 48.466% using the feature level fusion approach, and an HTER of 46.201% and ACER of 51.037% using the score level fusion approach, as shown in Table 12. These detection errors are very high compared to those reported in Section 5.3.1 and Section 5.3.2. Based on these results, we conclude that the cross-dataset classification is still challenging and needs to be addressed in future work. In addition, the detection model trained on the CASIA dataset performs better than that trained on the Replay-mobile dataset. The reason is that the CASIA dataset contains more general attack methods than the Replay-mobile dataset, as mentioned in Section 5.2. As a result, the classification model trained on CASIA dataset works as a more general case compared to the model trained on Replay-mobile dataset. This result suggests that we can obtain an efficient face-PAD model by collecting maximum data that can simulate all possible kinds of attacking methods.

As the final experiment, we compared the detection performance of our proposed method with a previous study conducted by Peng et al. [53] for cross-dataset testing. In the study by Peng et al. [53], they used two methods for image feature extraction, i.e., a combination of LBP and the guided scale LBP (GS-LBP) and local guided binary pattern (LGBP). A detailed comparison is provided in Table 13. As shown in this table, the study by Peng et al. produced errors of 41.25% and 51.29% for the use of LBP+GS-LBP and the LGBP feature extraction methods, respectively, in the case of using the CASIA dataset for training and Replay-mobile dataset for testing. Using our proposed method, we obtained an error of 12.459%, which is much smaller than that produced in the study by Peng et al. [53]. For the second case of using the Replay-mobile dataset for training and CASIA dataset for testing, our proposed method produced an error of 42.785%. Although this error is very high, it is still lower than 48.59% and 50.04% produced by the study by Peng et al. for the case of using LBP+GS-LBP and LGBP, respectively. In addition, we performed experiments for the face-PAD system based on deep features extracted by the CNN method to evaluate the influence of stacked CNN-RNN architecture on learning temporal information over the CNN architecture with the cross-dataset. Using the deep image features extracted by the CNN method [27], we obtained an error rate (HTER) of 21.496% for the case of using the CASIA dataset for training and the Replay-mobile dataset for testing. In the opposite way, we obtained an error of 34.530% for the case of using the Replay-mobile dataset for training and the CASIA dataset for testing. As shown in Table 13, our proposed method outperforms the face-PAD system based on CNN [27] in the case of using the CASIA dataset for training and the Replay-mobile dataset for testing with an error of 12.459% versus 21.496%. However, the error produced by our method is higher than that of the CNN-based method. The reason for this is that the CASIA dataset contains more general presentation attack methods than the Replay-mobile dataset. Therefore, although the detection model works well on the Replay-mobile dataset, it performs poorly in the CASIA dataset. Through these comparisons, we conclude that our proposed method outperforms the previous work conducted by Peng et al. [53] for the cross-dataset setup. In addition, we see that we should collect data from as many as possible presentation attack methods for training to ensure the PAD performance in the cross-dataset testing scenario.

## 6. Conclusions

In this study, we proposed a new approach for detecting presentation attack face images to enhance the security level of a face recognition system. The main idea of our study was the use of a very deep stacked CNN-RNN network to learn the discrimination features from a sequence of face images. The success of this network is offered by the use of a deep CNN network to efficiently learn and extract texture features of face images, and an LSTM (a special kind of RNN) to learn the temporal information from a sequence of face images. In addition, we confirmed that the combination of deep and handcrafted image features is sufficient for enhancing the performance of the detection system. Through our intensive experiments with two public datasets, i.e., the CASIA and Replay-mobile, we demonstrated a state-of-the-art detection performance compared with previous studies. We obtained an error rate of 1.286% using the CASIA dataset that contains 600 video files, and an error of 0.000% using the Replay-mobile dataset that contains 1030 video files of real and presentation attacks.

## Figures and Tables

**Table 1:** A summary of previous studies on face-PAD with comparison with our proposed method.

Category	Detection Method	Strength	Weakness
Uses still images	- Uses handcrafted image features [15,16,17,18,19,21,22,23,24,25]	- Detection system is simple and easy to implement - Can achieve high processing speed	- Detection performance is limited because of handcrafted features designed by humans based on limited observation aspects of face-PAD problem
	- Uses deep image features: CNN [20,26]	- Uses deep features extracted by CNN for enhancing detection performance	- More complex and requires more power and processing time than the methods that only use handcrafted image features
	- Uses a combination of deep and handcrafted image features [27]	- Uses a very deep CNN network to efficiently extract image features - Uses SVM for classification instead of fully-connected layer that might reduce the overfitting problem. - Higher detection performance using a combination of deep and handcrafted image features	- More complex and requires more power and processing time than the methods that only use handcrafted image features
Uses sequence images	- Uses stacked CNN-RNN network to learn the temporal relation between image frames for face-PAD [28]	- Obtains higher detection performance than previous methods that only use a still image for detection using information learnt from more than one image	- Complex structure requiring more power and processing time - The CNN network is shallow with only two convolution layers and one fully connected layer
	- Uses very deep stacked CNN-RNN to learn the temporal relation between image frames - Combines deep and handcrafted image features to enhance the detection performance (Proposed method)	- Uses very deep CNN network to efficiently extract image features for inputs of RNN - Obtains higher detection performance than previous methods using very deep CNN-RNN network and handcrafted image features	- Requires more power and processing time to process a sequence of images

**Table 2:** Detailed description of architecture of the stacked CNN-RNN network in our study.

Repeat Times	Layer Type	Padding Size	Stride	Filter Size	Number of Filters (Neurons)	Size of Feature Maps	Number of Parameters
1	Input Layer	n/a	n/a	n/a	n/a	5 × 224 × 224 × 3	0
2	Convolution	1 × 1	1 × 1	3 × 3	64	5 × 224 × 224 × 64	38,720
	ReLU	n/a	n/a	n/a	n/a	5 × 224 × 224 × 64	0
1	Max Pooling	n/a	2 × 2	2 × 2	1	5 × 112 × 112 × 64	0
2	Convolution	1 × 1	1 × 1	3 × 3	128	5 × 112 × 112 × 128	221,440
	ReLU	n/a	n/a	n/a	n/a	5 × 112 × 112 × 128	0
1	Max Pooling	n/a	2 × 2	2 × 2	1	5 ×56×56× 128	0
4	Convolution	1 × 1	1 × 1	3 × 3	256	5 ×56×56× 256	2,065,408
	ReLU	n/a	n/a	n/a	n/a	5 × 56 × 56 × 256	0
1	Max Pooling	n/a	2 × 2	2 × 2	1	5 × 28 × 28 ×256	0
4	Convolution	1 × 1	1 × 1	3 × 3	512	5 × 28 × 28 × 512	8,259,584
	ReLU	n/a	n/a	n/a	n/a	5 × 28 × 28 × 512	0
1	Max Pooling	n/a	2 × 2	2 × 2	1	5 × 14 × 14 × 512	0
4	Convolution	1 × 1	1 × 1	3 × 3	512	5 × 14 × 14 × 512	9,439,232
	ReLU	n/a	n/a	n/a	n/a	5 × 14 × 14 × 512	0
1	Max Pooling	n/a	2 × 2	2 × 2	1	5 × 7 × 7 × 512	0
1	Global Average Pooling	n/a	n/a	n/a	1	5 × 512	0
1	Fully Connected Layer	n/a	n/a	n/a	1024	5 × 1024	525,312
1	Batch Normalization	n/a	n/a	n/a	n/a	5 × 1024	4096
1	ReLU	n/a	n/a	n/a	n/a	5 × 1024	0
1	LSTM	n/a	n/a	n/a	n/a	1024	8,392,704
1	Dropout	n/a	n/a	n/a	n/a	1024	0
1	Fully Connected Layer	n/a	n/a	n/a	2	2	2050
Total number of parameters: 28,948,546 Total number of trainable parameters: 28,946,498 Total number of non-trainable parameters: 2048							

**Table 3:** Parameters of SGD method for training the stacked CNN-RNN network in our experiments.

Mini-Batch Size	Initial Learning Rate	Learning Rate Drop Period (Epochs)	Learning Rate Drop Factor	Number of Training Epochs	Momentum
4	0.00001	2	0.1	9	0.9

**Table 4:** Description of the CASIA dataset used in our study (unit: image sequences).

CASIA Dataset	Training Dataset (20 people)		Testing Dataset (30 people)		Total
	Real Access	Presentation Attack	Real Access	Presentation Attack	
Video	60	180	90	270	600
Image Sequence without Data Augmentation	10,940	34,148	16,029	49,694	110,811
Image Sequence with Data Augmentation	65,640	68,296	16,029	49,694	199,659

**Table 5:** Description of the Replay-mobile dataset used in our study (unit: image sequences).

Replay-Mobile Dataset	Training Dataset (12 people)		Validation Dataset (16 people)		Testing Dataset(12 people)		Total
	Real Access	Presentation Attack	Real Access	Presentation Attack	Real Access	Presentation Attack	
Video	120	192	160	256	110	192	1030
Image Sequence without Data Augmentation	35,087	56,875	47,003	75,911	32,169	56,612	303,657
Image Sequence with Data Augmentation	105,261	113,750	141,009	151,822	32,169	56,612	600,623

**Table 6:** Detection errors (APCER, BPCER, ACER, and HTER) of our proposed method with the CASIA dataset using three types of PAI (unit: %).

PAI	Wrap-photo Access			Cut-photo Access			Video Display			Overall			
	APCER	BPCER	ACER	APCER	BPCER	ACER	APCER	BPCER	ACER	APCER	BPCER	ACER	HTER
Using CNN Features [27]	3.975	2.770	3.373	0.643	2.770	1.7065	1.810	2.770	2.290	3.975	2.770	3.373	2.536
Using CNN-RNN Features	1.531	1.385	1.458	0.331	1.385	0.858	0.831	1.385	1.108	1.531	1.385	1.458	0.954
Using MLBP Features	9.133	10.343	9.738	10.018	10.343	10.181	9.425	10.343	9.884	10.018	10.343	10.181	9.488
FLF	3.508	0.917	2.212	0.676	0.917	0.797	1.292	0.917	1.104	3.508	0.917	2.212	1.443
SLF	1.536	1.036	1.286	0.507	1.036	0.771	0.121	1.036	0.579	1.536	1.036	1.286	0.910

**Table 7:** Description of subsets of the CASIA dataset used in our study (unit: image sequences).

Dataset Name	Training Dataset (20 people)		Testing Dataset (30 people)		Total
	Real Access	Presentation Attack	Real Access	Presentation Attack	
Low Quality Dataset	3140	11,019	5298	16,166	35,623
Normal Quality Dataset	3223	11,275	4949	16,141	35,588
High Quality Dataset	4577	11,854	5782	17,387	39,600
Wrap-Photo Dataset	10,940	12,871	16,029	19,271	59,111
Cut-Photo Dataset	10,940	9499	16,029	14,784	51,252
Video Display Dataset	10,940	11,778	16,029	15,639	54,386

**Table 8:** Detection errors (ACERs) of various face-PAD methods using a subset of the CASIA dataset according to the quality and type of presentation attack samples (unit: %).

Detection Method	Quality of the Presentation Attack Samples			Type of Presentation Attack Samples		
	Low Quality Dataset	Normal Quality Dataset	High Quality Dataset	Wrap-Photo Dataset	Cut-Photo Dataset	Video Access Dataset
Baseline Method [13]	13.0	13.0	26.0	16.0	6.0	24.0
IQA [18]	31.7	22.2	5.6	26.1	18.3	34.4
LBP-TOP [24]	10.0	12.0	13.0	6.0	12.0	10.0
LBP + Fisher Score + SVM [22]	7.2	8.8	14.4	12.0	10.0	14.7
Patch-based Classification [18]	5.26	6.00	5.30	5.78	5.49	5.02
LBP of Color Texture Image [19]	7.8	10.1	6.4	7.5	5.4	8.4
CNN + MLBP [27]	1.834	3.950	2.210	2.054	0.545	4.835
Proposed Method (FLF)	2.096	3.354	1.484	1.886	0.425	1.611
Proposed method (SLF)	1.417	0.040	1.085	2.005	0.428	1.423

**Table 9:** Comparison of detection error (ACER) of our proposed method with various previous studies (unit: %).

Baseline Method [13]	LBP + Fisher Score + SVM [22]	LBP of Color Texture Image [19]	Dynamic Local Ternary Pattern [23]	Patch-based Classification [18]	CNN + MLBP [27]	Proposed Method
17.000	13.100	6.200	5.400	5.070	1.696	1.286

**Table 10:** Detection errors (APCER, BPCER, ACER, and HTER) of our proposed method with the Replay-mobile dataset using two types of PAI (unit: %).

PAI	EER	Matte-Screen Attack			Print Attack			Overall			
		APCER	BPCER	ACER	APCER	BPCER	ACER	APCER	BPCER	ACER	HTER
Using CNN Features [27]	0.067	0.000	0.009	0.0045	0.000	0.009	0.0045	0.000	0.009	0.0045	0.0045
Using CNN-RNN Features	0.002	0.000	0.003	0.0015	0.000	0.003	0.0015	0.000	0.003	0.0015	0.0015
Using only MLBP features	4.659	8.820	1.937	5.379	2.451	1.937	2.194	8.820	1.937	5.379	5.684
FLF	0.000	0.000	0.000	0.000	0.000	0.000	0.000	0.000	0.000	0.000	0.000
SLF	0.000	0.000	0.003	0.0015	0.000	0.003	0.0015	0.000	0.003	0.0015	0.0015

**Table 11:** Detection results (APCER, BPCER, ACER, and HTER) of cross-dataset testing (Trained with CASIA; Tested with Replay-mobile) (unit: %).

PAI	Matte-Screen Attack			Print Attack			Overall			
	APCER	BPCER	ACER	APCER	BPCER	ACER	APCER	BPCER	ACER	HTER
FLF	4.304	22.714	13.509	0.039	22.714	11.377	4.304	22.714	13.509	12.459
SLF	12.838	34.341	23.589	0.822	34.341	17.581	12.838	34.341	23.589	20.632

**Table 12:** Detection results (APCER, BPCER, ACER, and HTER) of cross-dataset testing (Trained with Replay-mobile; Tested with CASIA) (unit: %).

PAI	Wrap-photo Attack			Cut-photo Attack			Video Attack			Overall			
	APCER	BPCER	ACER	APCER	BPCER	ACER	APCER	BPCER	ACER	APCER	BPCER	ACER	HTER
FLF	65.451	14.499	39.975	82.434	14.499	48.466	67.255	14.499	40.877	82.434	14.499	48.466	42.785
SLF	77.510	13.039	45.275	89.035	13.039	51.037	72.505	13.039	42.772	89.035	13.039	51.037	46.201

**Table 13:** Comparison of detection error (HTER) produced by our proposed method with the previous study by Peng et al. for the cross-dataset setup (unit: %).

Detection Method	Trained with	Tested with	HTER
Using LBP + GS-LBP [53]	CASIA	Replay-mobile	41.25
	Replay-mobile	CASIA	48.59
Using LGBP [53]	CASIA	Replay-mobile	51.29
	Replay-mobile	CASIA	50.04
Using CNN [27]	CASIA	Replay-mobile	21.496
	Replay-mobile	CASIA	34.530
Our Proposed Method	CASIA	Replay-mobile	12.459
	Replay-mobile	CASIA	42.785
